# Induction of Epithelial Mesenchimal Transition and Vasculogenesis in the Lenses of Dbl Oncogene Transgenic Mice

**DOI:** 10.1371/journal.pone.0007058

**Published:** 2009-09-16

**Authors:** Paolo Fardin, Marzia Ognibene, Cristina Vanni, Amleto De Santanna, Luigi Varesio, Alessandra Eva

**Affiliations:** 1 Laboratorio di Biologia Molecolare, Istituto G. Gaslini, Genova, Italy; 2 Sezione di Istologia, Dipartimento di Medicina Sperimentale, Università di Genova, Genova, Italy; Karolinska Institutet, Sweden

## Abstract

**Background:**

The Dbl family of proteins represents a large group of proto-oncogenes involved in cell growth regulation. The numerous domains that are present in many Dbl family proteins suggest that they act to integrate multiple inputs in complicated signaling networks involving the Rho GTPases. Alterations of the normal function of these proteins lead to pathological processes such as developmental disorders and neoplastic transformation. We generated transgenic mice introducing the cDNA of Dbl oncogene linked to the metallothionein promoter into the germ line of FVB mice and found that onco-Dbl expression in mouse lenses affected proliferation, migration and differentiation of lens epithelial cells.

**Results:**

We used high density oligonucleotide microarray to define the transcriptional profile induced by Dbl in the lenses of 2 days, 2 weeks, and 6 weeks old transgenic mice. We observed modulation of genes encoding proteins promoting epithelial-mesenchymal transition (EMT), such as down-regulation of epithelial cell markers and up-regulation of fibroblast markers. Genes encoding proteins involved in the positive regulation of apoptosis were markedly down regulated while anti-apoptotic genes were strongly up-regulated. Finally, several genes encoding proteins involved in the process of angiogenesis were up-regulated. These observations were validated by histological and immunohistochemical examination of the transgenic lenses where vascularization can be readily observed.

**Conclusion:**

Onco-Dbl expression in mouse lens correlated with modulation of genes involved in the regulation of EMT, apoptosis and vasculogenesis leading to disruption of the lens architecture, epithelial cell proliferation, and aberrant angiogenesis. We conclude that onco-Dbl has a potentially important, previously unreported, capacity to dramatically alter epithelial cell migration, replication, polarization and differentiation and to induce vascularization of an epithelial tissue.

## Introduction

The Dbl protein is the prototype member of a large family of guanine nucleotide exchange factors (GEFs) for Rho GTPases [1]–[3], which are known to regulate various physiological processes including actin cytoskeleton organization, cell movement, cell proliferation, cytokinesis, and apoptosis [4]–[6]. Consistent with the wide spectrum of actions of Rho-like proteins on growth regulation, Dbl and most Dbl-like proteins possess oncogenic potential. Oncogenic activation of Dbl occurs by truncation of the amino-terminal 497 residues [7], resulting in constitutively active carboxyl-terminal sequences that include a Dbl homology (DH) domain in tandem with a pleckstrin homology (PH) domain, the conserved motifs of the Dbl family. Dbl-mediated generation of transformed foci of NIH3T3 cells is probably caused by altered gene expression. Hence, Dbl was found to activate JNK and to stimulate transcription from NF-kB responsive elements and cyclin D1 promoter [8]–[10]. However, an attempt to produce Dbl-induced neoplastic transformation in transgenic mice failed to generate tumors [11]. In those studies we generated transgenic mice by introducing the entire Dbl transforming genomic sequences and the onco-Dbl cDNA linked to a set of different tissue specific promoters into the germ line of FVB/N mice. We reported that mice with the crystallin promoter, cry-*dbl*, the metallothionein promoter, MT-*dbl*, and a cosmid clone constructs, cos-*dbl*, expressed the Dbl protein in the lenses and developed cataracts [11].

A normal lens is characterized by a single layer of cells, the lens epithelium, which cover the anterior part of the lens that faces the cornea. The lens epithelium ends on the rims of the anterior surface of the lens and contains cells in the central region that do not divide, and are essentially quiescent, surrounded by a dividing zone of cells followed at the equatorial fringe by the dividing cells that differentiate into fiber cells. The fiber cells mass provides the lens with its functional phenotype, transparency, while the epithelial cells are the metabolically active cells of the lens and sustain the physiological health of this tissue. The lens epithelium is recognizable as a morphological entity early during gestation and remains in this morphological state for the rest of the life.

The cataracts of the Dbl transgenic mice were characterized by a dramatic fibroblastic dysplasia of the lens, disruption of the lens architecture and aberrant epithelial cell proliferation. The eyes of adult cry-*dbl* transgenic mice were the ones with milder pathological alterations. Specifically, the embryonic nucleus of the lens was typically intact while the posterior region of the lens, which is normally occupied by the elongating secondary fiber cells, showed extensive vacuolization. In addition, the pattern of differentiation of the secondary fiber cells at the equatorial region was abnormal, and there was evidence of disorganization in the cuboidal epithelial cells. The dysplasia observed in the cos-*dbl* family with cataracts was very similar to that seen in the MT-*dbl* lenses, but occurred more slowly than that of the eyes of MT-*dbl* mice. Differentiation of the secondary fiber cells was abnormal, showing posterior migration of equatorial epithelial cells and defects in nuclear resorption. Anterior epithelial cells became disorganized and multilayered, forming epithelial plaques lying underneath the lens capsule. The dysplastic epithelial cells proliferated and dispersed throughout the cortex of the lens and subsequently adopted the appearance and behavior of fibroblastic cells. The nuclei became elongated, irregular in shape, and surrounded by connective tissue.

We have now analyzed in depth the alterations caused by the expression of Dbl oncogene in the mouse lens epithelial cells. We have chosen the transgenic mouse family expressing the onco-Dbl cDNA linked to the metallothionein promoter because it shows the most dramatic dysplasia of the lens and thus it probably represents the animal model where Dbl more strongly interferes with the differentiation and growth of lens cells.

We report here the transcriptome analysis of wt and MT-*dbl* transgenic mouse lenses at different time points after birth. The results obtained indicate down-regulation of epithelial cell markers and up-regulation of fibroblast markers, compatible with an EMT event [12]–[14]. Moreover, genes encoding proteins involved in the positive regulation of apoptosis were heavily down regulated while those involved in the inhibition of apoptosis were strongly upregulated, indicative of evasion from cell death. Finally, several genes encoding proteins involved in the process of vascularization were upregulated. Hystological examination confirmed that lymphangiogenesis and angiogenesis occur in the Dbl transgenic lenses.

## Materials and Methods

### DNA construct and generation of transgenic mice

The construction of the plasmid containing the Dbl oncogene cDNA under the control of the MT promoter and the generation of the transgenic mice was previously described [11]. Briefly, the mouse MT promoter construct, P341-3, kindly provided by P. Howley [15], was cleaved at the unique BglII site localized 3′ of the MT promoter and ligated to the BamHI site of the Dbl cDNA [16]. Embryos at the single-cell stage were isolated from superovulated FVB/N females mated to FVB/N males. Transgenic mice were generated by pronuclear microinjection of the DNA construct at a concentration of 2 mg/ml in 10 mM Tris and 0.1 mM EDTA, pH 7.8. Embryos were reimplanted into pseudopregnant ICR/Hsd foster mothers and allowed to develop to term. Mice found to carry the transgene and to transmit it to progeny were cross bred to Balb/c mice, which are rd+.

### Microarray experiments and statistical analysis

Whole lenses were removed from 2, 14, and 42 day old Dbl transgenic and wild type mice and stored in RNAlater RNA stabilizing reagent (Qiagen, Hilden, Germany). 4 to 8 individual lenses were pooled and homogenized in buffer through a 22-gauge needle. Three independent pools from each time and condition were used as replicates. Total RNA was isolated using RNeasy mini kit (Qiagen) according to the manufacturer's instructions. The quality of RNA was evaluated using Agilent Bioanalyzer 2100 (Agilent Technologies, Germany) and the RNA was quantified by NanoDrop (NanoDrop Technologies Wilmington, USA).

For the 2-, 14- and 42-days samples, 10 µg of each RNA sample was reverse-transcribed into cDNA and biotin labeled according to the Affymetrix's instructions (Affymetrix, SantaClara, CA). Biotin-labeled cRNA was cleaned with the RNeasy Mini Kit (Qiagen) and ethanol precipitation, analyzed for quality with Agilent Bioanalyzer 2100 and fragmented by incubation at 94°C for 35 min in 40 mM Tris-acetate, pH 8.1, 100 mM potassium acetate, 30 mM magnesium acetate. Fragmented cRNA was used for hybridization to Affymetrix Murine Genome Array U74Av2. GeneChips were scanned using an Affymetrix GeneChip Scanner 3000. All microarrays were examined for surface defects, grid placement, background intensity, housekeeping gene expression, and a 3′∶5′ ratio of probe sets from genes of various lengths.

The complete data set for each microarray experiments has been deposited in the Gene Expression Omnibus public repository at National Center for Biotechnology Information (accession number GSE15694).

Expression values were quantified and array quality control was performed with the statistical algorithms implemented in Affymetrix Microarray Suite 5.0. The resulting data were analyzed by GeneSpring Expression Analysis Software Gx 7.3 (Silicon Genetics, Redwood City, CA). After normalization process, the gene expression levels of replicate experiments were averaged and only genes that were modulated by at least 1.5-fold in the Dbl transgenic lenses relative to the wild type lenses (means of three experiments) were considered differentially expressed. The significance of gene expression differences between the two experimental conditions was calculated using a non-parametric test (Wilcoxon-Mann-Whitney U test, p-value cutoff 0.05).

The gene lists were analyzed and clustered in different pathways or functional categories sorted by a p-value<0.05, according to their biological function using the Database for Annotation, Visualization and Integrated Discovery 2.0 (DAVID 2.0) tool [17].

### RT-PCR validation

For the 2, 14, and 42 day old samples 2 µg of total RNA was reverse-transcribed using Advantage RT-PCR KIT (Clontech, Mountain View, CA) and Real Time quantitative PCR (qRT-PCR) was performed on a Prism 7500 Sequence Detection System (Applied Biosystems, Inc. [ABI], Foster City), using SYBR Green PCR Master Mix (Applied Biosystems), and 300 nM sense and antisense oligonucleotide primers (TIBMolbiol, Italy and Qiagen). Primers for crystallin α A (Cryaa) and crystallin γ f (Crygf) were purchased from Qiagen (Quantitect primer assay, codes 12954 and 12969 respectively). All other primer pairs (Table S1) were designed using Primer-3 software [18] from sequences in GenBank with a Tm optimum of 60°C and a product length of 80–150 nt and tested before use to confirm appropriate product size and optimal concentrations. qRT-PCR was conducted in triplicate for each target transcript under the following cycling conditions: initial denaturation of 3 min during which the well factor was measured, 50 cycles of 15 s at 95°C followed by 30 s at 60°C. Fluorescence was measured during the annealing step in each cycle. Expression data were normalized on the values obtained in parallel for three reference genes (glutathione peroxidase 1 (Gpx1); dynactin 2 (Dctn2); and presenilin 1 (Psen1)) using the Bestkeeper software [19]. Relative expression values and standard error (SE) were calculated using Q-gene software [20].

### Histochemical and immunohistochemical analysis

Eyes from 2, 14, 28, 42 day old mice were fixed with 2% paraformaldehyde and paraffin embedded. For histological analysis five micrometer thick serial sections were stained with Ignesti (aurantia, emallume, methyl blue, orange G, and phosphomolybdic acid) or Azan-Mallory. For immunohistochemistry analysis serial sections were treated in a microwave oven four times with citrate buffer (pH 6.0) for 5 min at 960 W. Sections were saturated with 10% BSA in PBS with 0.1% Triton X-100 and incubated overnight at 4°C in a humidified chamber with the specific primary Ab. For immunohistochemical analyses anti MMP12 rabbit monoclonal (Novus Biologicals), anti E-cadherin mouse monoclonal [21], anti Ccl2/MCP-1 rabbit polyclonal (Abcam), anti Bcl2A1 rabbit polyclonal (Abcam), and SPP-1 mouse monoclonal (Santa Cruz Biotechnology) antibodies were used. The reactions were developed with LSAB2 System-HRP (Dako) after addition of a secondary goat anti-mouse antiserum (DAKO). Images were obtained with a Leica DMRB microscope. Micrographs were taken on LEICA DFC 320 camera.

### TUNEL Assay

DNA cleavage was assessed by enzymatic end-labeling of DNA strand breaks with a commercial kit (In Situ Cell Death Detection kit, Fluorescent, Roche Molecular Chemicals). Labeling was carried out according to the manufacturer's instructions. Briefly, paraffin embedded lens sections were deparaffined and rehydratated and washed with deionized water. Sections were, then, permeabilized with 0.1% Triton X-100 for 8 minutes at room temeprature. After rinsing with PBS, slides were incubated with 20 µL of terminal deoxynucleotidyl transferase (TdT)-mediated dUTP nick end labeling (TUNEL) reaction mixture, containing TdT- and FITC-labeled dUTP, in a humidified atmosphere for 1 hour at 37°C in the dark. Rinsed slides were then coverslipped with Vectashield mounting medium containing 4′,6′-diamidino-2-phenylindole (DAPI; Vector Laboratories, Burlingame, CA) for nuclear counterstaining. TUNEL+ apoptotic cells, which fluoresce bright green, were viewed with a Nikon Eclipse E1000 fluorescent microscope (Nikon Corp., Tokyo, Japan) equipped with a FITC filter.

## Results

**Figure 1:**
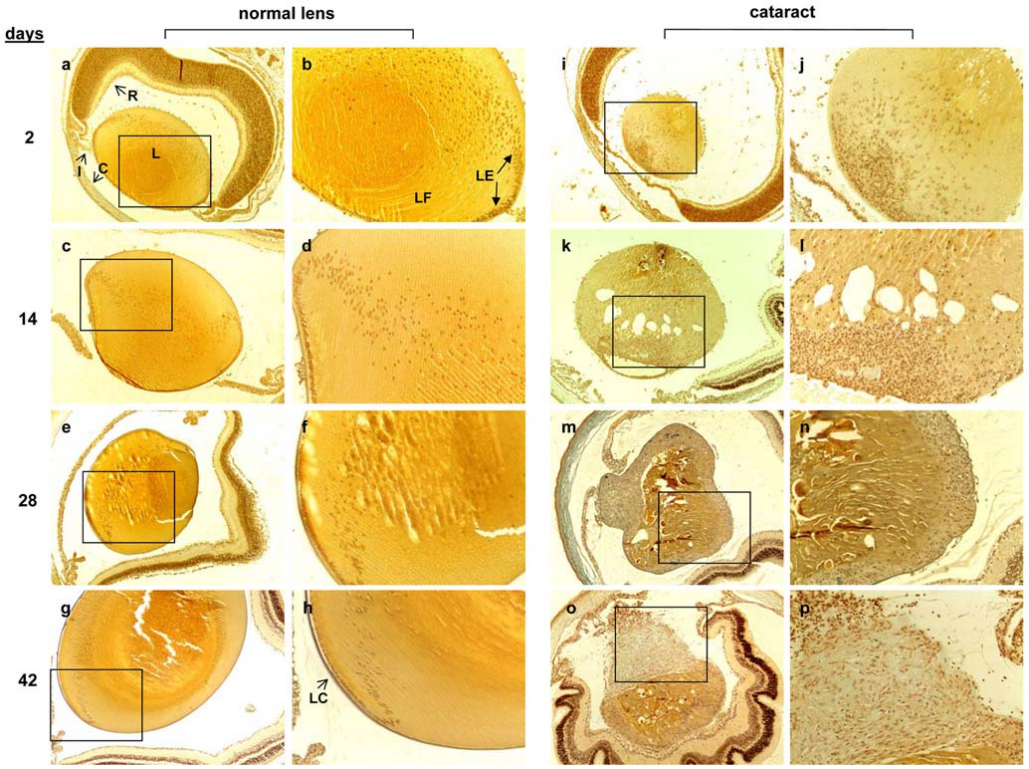
Developmental progression of Dbl transgenic lenses. Eyes were fixed with 2% paraformaldehyde and paraffin embedded. Serial sections were stained with Ignesti. (a, h,) normal lenses; (i–p) lenses from transgenic mice. (a, b, i, j) lenses from 2 day old mice; (c, d, k, l) lenses from 14 day old mice; (e, f, m, n) lenses from 28 day old mice; (g, h, o,p) lenses from 42 day old mice. (a, c, e, g, i, k, m, o) 40×; (b, d, f, h, j, l, n, p) 100×. The boxes in a, c, e, g, i, k, m, and o denote the regions shown at higher magnification in b, d, f, h, j, l, n, and p, respectively. Symbols: C, cornea; I, iris; L, lens; R, retina; LC, lens capsule; LE, lens epithelial cell monolayer; LF, lens fiber cells.

Mice carrying the Dbl cDNA linked to the mouse metallothionein promoter express the Dbl protein in the lenses and show dominant bilateral lens cataracts characterized by disruption of the lens architecture, aberrant epithelial cell proliferation and a dramatic fibroblastic dysplasia [11]. Fig. 1 shows the histology of Dbl transgenic mouse lenses in comparison with normal nontransgenic ones at different times after birth. The normal lens remains morphologically the same and only grows in size as the mouse grows older (Fig. 1 a–h). At higher magnification it is clearly visible the migration at the equatorial region of the epithelial cells and their differentiation into lens fiber cells. Transgenic mice, on the other hand, showed disruption of the lens architecture and epithelial cells exhibited a disorganized proliferation. At the equatorial region, the epithelial cells migrate towards the anterior as well as the posterior region and disperse throughout the lens, adopted the appearance of fibroblastic cells, with spindle-shaped nuclei (Fig. 1, m–p). Fiber cells could no longer be observed indicating abnormalities in the differentiation process of lens cells (Fig. 1, i–p). By 6 weeks of age the dysplasia of the lens was extensive accompanied by dramatic changes in the other parts of the eye. The iris appeared disorganized and attached to the anterior part of the lens and the retina appeared disorganized and convoluted (Fig. 1, o). Moreover, the lens capsule appeared partially destroyed (Fig. 1, p).

To further analyze the alterations caused by the expression of onco-Dbl in the mouse lens epithelial cells (Dbl-lens), we analyzed the gene transcriptional profile of the lenses of the transgenic mice using the Affymetrix Murine Genome Array U74Av2 chip. RNA was extracted from 2, 14 and 42 day old Dbl transgenic and wild type mice lenses. Three independent RNA pools for each experimental condition were analyzed. The genes modulated by more than 1.5 fold in Dbl transgenic lenses relative to wild type samples were considered and the significance of gene expression differences between the two experimental conditions was calculated using a non-parametric test (Wilcoxon-Mann-Whitney U test) with a confidence level of 95%. We identified a total of 2,776 genes differentially expressed in Dbl transgenic mice at 2, 14 and 42 day old lenses. Analysis of the biological processes was performed to assess the overall number of significantly changed genes in various functional categories according to Gene Ontology (GO) annotations [22] and to determine the general trend of the molecular response to Dbl expression. The transcriptional profile induced by onco-Dbl was mainly related to changes in morphogenesis, cell cycle, cell adhesion, cell proliferation, signaling cascade, nucleic acid and protein metabolism, apoptosis, and angiogenesis (Table 1).

**Table 1:** Functional analysis of genes induced or suppressed in Dbl transgenic mouse^a^.

GO ID^b^	GO Category	Genes	%^c^	P-value^d^
**Induced genes**				
GO:0006396	RNA processing	50	2.91%	1.08E-07
GO:0007049	cell cycle	80	4.66%	2.14E-06
GO:0046907	intracellular transport	76	4.42%	1.73E-04
GO:0006915	apoptosis	61	3.55%	1.91E-04
GO:0007399	nervous system development	60	3.49%	2.30E-04
GO:0007242	intracellular signaling cascade	99	5.76%	2.76E-04
GO:0000902	cellular morphogenesis	47	2.74%	1.13E-03
GO:0006281	DNA repair	24	1.40%	2.79E-03
GO:0006954	inflammatory response	23	1.34%	3.45E-03
GO:0009653	morphogenesis	91	5.30%	3.77E-03
GO:0006956	complement activation	10	0.58%	3.96E-03
GO:0009416	response to light stimulus	10	0.58%	3.96E-03
GO:0050770	regulation of axonogenesis	7	0.41%	1.09E-02
GO:0043066	negative regulation of apoptosis	17	0.99%	1.47E-02
GO:0008203	cholesterol metabolism	10	0.58%	2.00E-02
GO:0030182	neuron differentiation	28	1.63%	2.09E-02
GO:0006096	glycolysis	10	0.58%	2.23E-02
GO:0030029	actin filament-based process	19	1.11%	2.48E-02
GO:0006350	transcription	184	10.71%	2.52E-02
GO:0001568	blood vessel development	20	1.16%	2.56E-02
GO:0051028	mRNA transport	6	0.35%	3.52E-02
GO:0007155	cell adhesion	55	3.20%	3.52E-02
GO:0007219	Notch signaling pathway	8	0.47%	3.90E-02
GO:0006909	phagocytosis	7	0.41%	4.34E-02
GO:0051270	regulation of cell motility	8	0.47%	4.87E-02
**Suppressed genes**				
GO:0019538	protein metabolism	253	19.05%	2.47E-12
GO:0007423	sensory organ development	10	0.75%	3.52E-07
GO:0007422	peripheral nervous system development	12	0.90%	1.75E-06
GO:0009887	organ morphogenesis	55	4.14%	2.40E-06
GO:0042254	ribosome biogenesis and assembly	19	1.43%	6.47E-05
GO:0043065	positive regulation of apoptosis	22	1.66%	7.98E-05
GO:0001654	eye development	14	1.05%	2.66E-04
GO:0006066	alcohol metabolism	28	2.11%	3.67E-04
GO:0006412	translation	22	1.66%	4.19E-04
GO:0006082	organic acid metabolism	43	3.24%	9.38E-04
GO:0006629	lipid metabolism	47	3.54%	3.90E-03
GO:0006457	protein folding	24	1.81%	3.93E-03
GO:0006096	glycolysis	10	0.75%	4.28E-03
GO:0007399	nervous system development	43	3.24%	6.87E-03
GO:0046907	intracellular transport	53	3.99%	1.24E-02
GO:0006508	proteolysis	54	4.07%	1.36E-02
GO:0006915	apoptosis	42	3.16%	1.39E-02
GO:0006520	amino acid metabolism	22	1.66%	1.60E-02
GO:0048598	embryonic morphogenesis	15	1.13%	1.70E-02
GO:0046849	bone remodeling	11	0.83%	1.85E-02
GO:0007155	cell adhesion	45	3.39%	2.28E-02
GO:0007223	frizzled-2 signaling pathway	5	0.38%	2.49E-02
GO:0016055	Wnt receptor signaling pathway	12	0.90%	2.79E-02
GO:0008284	positive regulation of cell proliferation	13	0.98%	2.85E-02
GO:0007267	cell-cell signaling	24	1.81%	2.93E-02
GO:0007167	enzyme-linked receptor protein signaling	21	1.58%	3.72E-02
GO:0007179	TGF beta receptor signaling pathway	7	0.53%	3.84E-02
GO:0009308	amine metabolism	27	2.03%	4.07E-02
GO:0006979	response to oxidative stress	7	0.53%	4.22E-02

The Gene Ontology categories and the number of significantly changed genes in the various functional categories are listed. The categories are divided into induced genes (upper panel) and suppressed genes (lower panel) according to their differential expression in Dbl transgenic versus wild type lenses.

a GO analysis performed using NIH DAVID http://david.abcc.ncifcrf.gov.

b Gene ontology ID numbers obtained from AmiGO http://www.genedb.org/amigo-cgi/go.cgi.

c % of total induced or suppressed genes.

d T-test p-value (confidence of 95%) indicates the gene enrichment for that pathway.

Some GO biological process categories were selected for a more detailed analysis because more closely related to lens biology, such as eye development, Wnt and TGFβ pathway, and to oncogene-induced cell transformation, such as cell adhesion, apoptosis, blood vessel development and Notch pathway. For each category we considered the genes that were modulated by more than 1.5 fold in at least two time points out of three. As shown in Table 2, there is a strong decrease in RNA levels of genes encoding lens structural proteins and lens-fiber specific markers, in all the time points, indicating that Dbl strongly perturbs lens structure and function. Perturbation of epithelial cell features, migration and polarity characteristics are indicated by down regulation of laminin α2 (Lama2), collagen type IV (Col4a1, Col4a2 and Col4a3) and E-cadherin (Cdh1) and up-regulation of N-cadherin (Cdh2), collagen type 1 (Col1a1), periostin (Postn), matrix metalloproteinase 12 (Mmp12) and the transforming growth factor beta induced gene (Tgfbi), a structural homolog of periostin. Genes involved in the pro-apoptotic process are mainly down-regulated in Dbl transgenic lenses, whereas genes with anti-apoptotic activity are up-regulated. These data support a role for Dbl in promoting cell survival. Moreover, our results suggest that onco-Dbl may induce neovascularisation, since modulation of genes involved in angiogenesis such as Anxa2, Ctfg, Eng, Nrp, Plat, and mostly Ccl2, a potent inducer of angiogenesis [23] is also observed in Dbl lenses. Finally, onco-Dbl alters the gene expression profile of Notch, Wnt and TGFβ-mediated developmental signalling pathways, critical for the morphogenesis of many vital organs and tissues [24].

**Table 2:** Differentially expressed genes of selected functional category.

Function^a^	Gene Name	GenBank	Description	2 days^b^	14 days^b^	42 days^b^
Eye development	Bfsp1	AB003147	beaded filament structural protein in lens-CP94	−12.28	−20.80	−30.93
	Cryaa	J00376	crystallin, alpha A	−2.10	−2.16	−2.15
	Cryab	AI842724	crystallin, alpha B	−2.57	−3.31	−1.91
	Cryba1	AJ239052	crystallin, beta A1	−2.25	−1.97	−2.34
	Crybb2	M60559	crystallin, beta B2	−3.45	−2.89	−4.57
	Crygb	AV360800	crystallin, gamma B	−6.81	−11.09	−13.85
	Crygc	Z22574	crystallin, gamma C	−3.19	−5.14	−5.40
	Crygd	M16512	crystallin, gamma D	−5.46	−11.99	−5.00
	Crygf	X57855	crystallin, gamma F	−2.22	−3.65	−3.14
	Crygs	AF032995	crystallin, gamma S	−2.97	−2.71	−3.18
	Mip	U27502	major intrinsic protein of eye lens fiber	−5.41	−7.32	−11.43
Cell Adhesion/ECM	Catna2	D25282	catenin alpha 2	−2.39	−2.18	−2.80
	Cdh1	X60961	cadherin 1	−2.43	−1.16	−1.81
	Cdh2	M31131	cadherin 2	3.23	1.62	2.56
	Cdh5	AI853217	cadherin 5	−1.59	2.41	6.52
	Col1a1	U03419	procollagen, type I, alpha 1	3.01	10.66	5.05
	Col4a1	M15832	procollagen, type IV, alpha 1	−4.93	−2.00	−3.03
	Col4a2	X04647	procollagen, type IV, alpha 2	−5.15	−3.17	−3.86
	Col4a3	Z35166	procollagen, type IV, alpha 3	−8.39	−1.40	−3.77
	Lama2	U12147	laminin, alpha 2	−3.98	−1.73	−7.33
	Itga6	X69902	integrin alpha 6	−4.00	−1.72	−3.12
	Mmp12	M82831	matrix metalloproteinase 12	1.81	17.70	11.12
	Ncam1	X15052	neural cell adhesion molecule 1	2.18	1.18	1.81
	Postn	D13664	periostin, osteoblast specific factor	2.21	3.78	3.12
	Tgfbi	L19932	transforming growth factor, beta induced	4.65	9.86	18.48
	Timp1	V00755	tissue inhibitor of metalloproteinase 1	2.66	5.41	6.20
	Tnc	X56304	tenascin C	1.20	3.28	2.58
	Vcam1	U12884	vascular cell adhesion molecule 1	4.06	2.98	3.56
	Vcl	AI462105	vinculin	1.57	3.19	4.12
TGFβ signaling pathway	Acvrl1	Z31664	activin A receptor, type II-like 1	−1.77	−1.37	−2.03
	Atf3	U19118	activating transcription factor 3	3.16	2.90	2.08
	Bmp1	L24755	bone morphogenetic protein 1	1.61	2.57	2.73
	Bmpr1a	D16250	bone morphogenetic protein receptor, type 1A	2.09	2.11	10.27
	Ccl7	X70058	chemokine (C-C motif) ligand 7	5.05	10.32	1.63
	Ccl8	AB023418	chemokine (C-C motif) ligand 8	5.15	14.00	3.74
	Fgfr1	U22324	fibroblast growth factor receptor 1	2.05	2.51	2.12
	Inhbb	X69620	inhibin beta-B	8.41	1.88	7.41
	Pcdha4	D86916	protocadherin alpha 6	2.35	2.10	2.15
	Smad1	U58992	MAD homolog 1 (Drosophila)	−2.61	−4.97	−2.49
	Smad5	U58993	MAD homolog 5 (Drosophila)	−1.75	−2.58	−1.08
Wnt signaling pathway	Cldn5	U82758	claudin 5	5.12	1.52	5.05
	Fhos2	AA795285	formin-family protein FHOS2	3.93	3.12	3.84
	Foxl1	X92498	forkhead box L1	−2.20	−1.74	−2.48
	Frat1	U58974	frequently rearranged in advanced T-cell lymphomas	3.13	1.35	2.35
	Fzd2	AW123618	frizzled homolog 2	3.77	3.03	6.64
	Gngt2	AI882325	guanine nucleotide binding protein, gamma transducing activity polypeptide 2	22.54	2.35	9.32
	Nlk	AF036332	nemo like kinase	1.89	−1.09	2.68
	Sfrp1	U88566	secreted frizzled-related sequence protein 1	−3.80	−3.44	−3.63
	Tcf12	X64840	transcription factor 12	1.73	2.04	2.51
	Tcfap2c	X94694	transcription factor AP-2, gamma	2.70	1.63	1.82
	Tle3	X73360	transducin-like enhancer of split 3, homolog of Drosophila E	−1.60	−2.76	−1.63
	Wnt1	M11943	wingless-related MMTV integration site 1	−2.41	−1.41	−1.89
	Wnt10b	U61970	wingless related MMTV integration site 10b	−2.79	−1.61	−1.94
	Wnt6	M89800	wingless-related MMTV integration site 6	−2.94	−2.76	−2.98
Notch signaling pathway	Capg	X54511	capping protein, gelsolin-like	2.53	8.75	3.61
	Cntn1	X14943	contactin 1	1.79	2.19	2.40
	Dll1	X80903	delta-like 1 (Drosophila)	7.78	2.34	3.90
	Hdac5	AF006602	histone deacetylase 5	2.67	1.86	2.71
	Hdac6	AF006603	histone deacetylase 6	1.65	1.59	1.62
	Mfng	AF015769	manic fringe homolog	2.37	1.62	2.18
	Neurod4	AF036257	neurogenic differentiation 4	4.27	1.31	1.90
	Psen2	U57325	presenilin 2	1.92	1.43	1.54
Positive regulation of apoptosis	Bad	AV102186	Bcl-associated death promoter	2.06	1.12	−1.84
	Bclaf1	AA874446	BCL2-associated transcription factor 1	1.53	−2.36	−2.30
	Casp7	U67321	caspase 7	−6.29	−4.73	−4.08
	Casp9	AB019600	caspase 9	−1.87	−1.96	−1.72
	Cideb	AF041377	cell death-inducing DNA fragmentation factor, alpha subunit-like effector B	−5.13	−3.85	−2.32
	Dapk2	AB018002	death-associated kinase 2	−2.07	−1.74	−1.05
	Faf1	AV222925	Fas-associated factor 1	−1.73	−2.18	−1.60
	Tnfrsf6	M83649	tumor necrosis factor receptor superfamily, member 6	−17.51	−16.08	−27.61
Negative regulation of apoptosis	Bcl2a1c	U23778	hematopoietic-specific early-response A1-c protein	5.71	3.57	4.12
	Bcl2a1a	U23781	hematopoietic-specific early-response A1-a protein	3.49	7.35	8.53
	Bnip1	AW060311	BCL2/adenovirus E1B interacting protein 1, NIP1	1.21	1.87	2.38
	Bnip2	AF035207	BCL2/adenovirus E1B 19 kDa-interacting protein 1, NIP2	5.63	3.94	4.81
	Spp1	X13986	secreted phosphoprotein 1	68.23	57.16	25.84
Angiogenesis	Anxa2	M14044	annexin A2	1.86	1.73	2.70
	Ccl2	M19681	chemokine (C-C motif) ligand 2	56.25	67.27	29.15
	Cited2	Y15163	cbp/p300-interacting transactivator, with glu/asp-rich carboxy-terminal domain, 2	1.63	1.13	2.59
	Ctgf	M70642	connective tissue growth factor	3.21	2.66	1.94
	Eng	X77952	endoglin	1.27	2.41	2.55
	Nrp	D50086	neuropilin 1	1.82	2.33	4.16
	Plat	J03520	plasminogen activator, tissue	2.07	2.43	1.58
	Sema3a	D85028	sema domain, immunoglobulin domain (Ig), secreted, (semaphorin) 3A	−3.45	−1.67	−1.92

The differentially expressed genes and their relative fold changes are listed for each of the time points. The genes were divided according to their Gene Ontology functional categories.

a The common gene name, the genbank accession number, a brief gene description, and the fold change value are specified for each gene.

b Fold change is calculated as a ratio of Dbl/wt signals (average of expression level of three experiments). For down-regulated genes the ratio is expressed as the negative reciprocal.

During the first 6 weeks of examination, as differentiation and growth of the lens ensued, no major differences in the overall trend of gene modulation induced by Dbl expression were observed. Only in a few cases changes were seen. For example, vascular endothelial cadherin (Cdh5) was down regulated at 2 days, but became gradually up-regulated by two and six weeks of age, and Eng was basically unchanged at 2 days but became up-regulated at 2 and 6 weeks of age.

The data of the gene expression profile in Dbl lenses, such as up-regulation of genes involved in cell proliferation, down-regulation of lens-specific genes and of epithelial cell markers, and up-regulation of fibroblast markers are in agreement with the histological observations (Fig. 1). Moreover, modulation of genes such as cadherins and collagens type 1 and 4 suggested that Dbl oncogene induces changes in lens cells compatible with an EMT process. EMT requires loss of epithelial polarity and epithelial specific proteins, induction of a fibroblastoid phenotype and of mesenchymal markers, and acquisition of a migratory phenotype [12], [25], [26]. We searched the results obtained and determined a 21-transcript list of genes modulated by Dbl expression and involved in EMT (Table 3). An overlapping of 17 out of 20 genes exists with the genes in the GO categories of Table 2. The selected genes include hallmarks of EMT such as E-cadherin (Cdh1), N-cadherin (Cdh2), collagen 1 (Col1a1), collagen 4 (Col4a1, Col4a2, Col4a3), smooth muscle alpha-actin (Asma), and tight junction protein 1 (Zo-1) and genes whose modulation has been implicated in EMT processes such as inhibition of cell adhesion, cell–matrix interaction, disruption of basement membrane and digestion of extracellular matrix (laminin, Vil2, Itga6, Tgfbi, Plat, Mmp12), and inhibition of apoptosis, increased survival and proliferation (Casp7, Casp9, Bcl2a1, Ki-67). Representative references describing the involvement of these genes in EMT process are given in Table 3. These observations suggest that onco-Dbl expression in mouse lenses induces events associated with EMT.

**Table 3:** Differentially expressed genes related to the EMT process.

Gene name^a^	GenBank	Description	2 days^b^	14 days^b^	42 days^b^	References
α-Sma	X13297	actin, alpha 2, smooth muscle, aorta	3,96	1,85	1,06	[43], [44]
Bcl2a1c	U23778	hematopoietic-specific early-response A1-c	5,71	3,57	4,12	[45], [46]
Bcl2a1a	U23781	hematopoietic-specific early-response A1-a	3,49	7,35	8,53	[45], [46]
Bmp1	L24755	bone morphogenetic protein 1	1,61	2,57	2,73	[13]
Bmpr1a	D16250	bone morphogenetic protein receptor, type 1A	2,09	2,11	10,27	[47]
Casp7	U67321	caspase 7	−6,29	−4,73	−4,08	[48], [49]
Casp9	AB019600	caspase 9	−1,87	−1,96	−1,72	[50]
Cdh1	X60961	cadherin 1	−2,43	−1,16	−1,81	[51], [52]
Cdh2	M31131	cadherin 2	3,23	1,62	2,56	[53], [54]
Col1a1	U03419	procollagen, type I, alpha 1	3,01	10,66	5,05	[55]
Col4a1	M15832	procollagen, type IV, alpha 1	−4,93	−2	−3,03	[44], [56]
Col4a2	X04647	procollagen, type IV, alpha 2	−5,15	−3,17	−3,86	[44], [56]
Col4a3	Z35166	procollagen, type IV, alpha 3	−8,39	−1,4	−3,77	[44], [56]
Ki-67	X82786	antigen identified by monoclonal antibody Ki 67	4,56	5,3	17,18	[57], [58]
Itga6	X69902	integrin alpha 6	−4	−1,72	−3,12	[43], [44]
Lama2	U12147	laminin, alpha 2	−3,98	−1,73	−7,33	[44], [59]
Mmp12	M82831	matrix metalloproteinase 12	1,81	17,7	11,12	[60], [61]
Plat	J03520	plasminogen activator, tissue	2,07	2,43	1,58	[62]
Tgfbi	L19932	transforming growth factor, beta induced	4,65	9,86	18,48	[63]
Vil2	X60671	villin 2	−2,27	−1,64	−1,79	[63]
Zo-1	D14340	tight junction protein 1	−3,04	−2,14	−1,69	[64], [65]

The differentially expressed genes and their relative fold changes are listed for each of the time points.

a Common gene name, genbank accession number, a brief gene description, and the fold change value are specified for each gene.

b Fold change is calculated as a ratio of Dbl/wt signals (average of expression level of three experiments). For down-regulated genes the ratio is expressed as the negative reciprocal.

**Figure 2:**
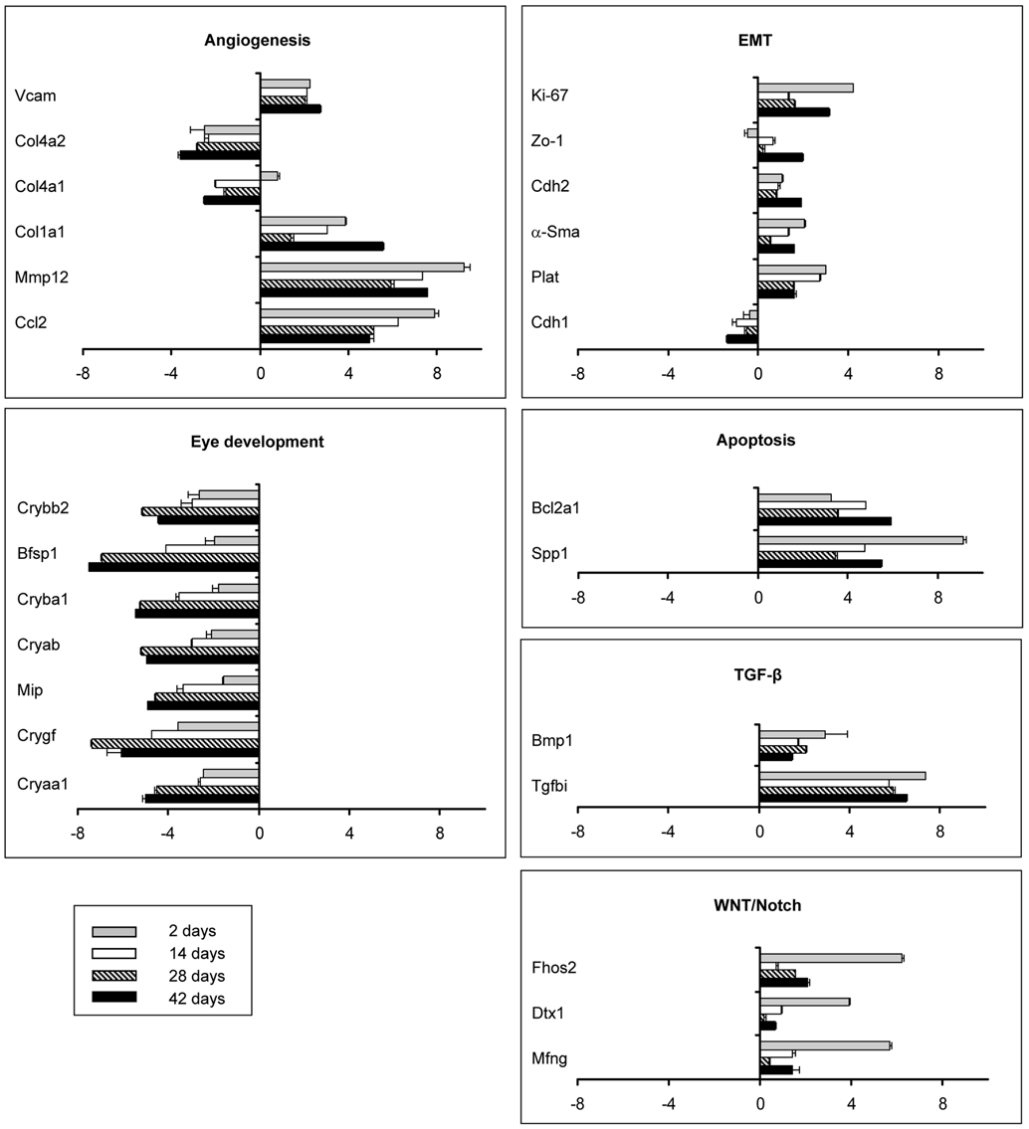
qRT-PCR analysis of genes selected from the microarray expression profile. qRT-PCR analysis of genes selected from the microarray expression profile. For the 2, 14, and 42 day old Dbl transgenic and wild type mice lenses 2 µg of total RNA was reverse-transcribed and Real Time quantitative PCR (qRT-PCR) was conducted in triplicate for each target transcript. Expression changes of 24 selected genes were evaluated in relation to the values obtained in parallel for three reference genes. The results are expressed as log2 ratios of fold changes (Dbl relative to wild type lenses) and the mean of triplicate determinations for each target transcript is shown. Positive values indicate that the mRNA level of a particular gene is up-regulated, whereas negative values indicate that the transcript is down-regulated. The bars represent SE.

To validate the microarray results, mRNA levels for a subset of genes were quantified by quantitative real time PCR (qRT-PCR) (Fig. 2). We found a 100% concordance between qRT-PCR and Affymetrix data with respect to the direction of the expression changes of all genes but Col4a1, for which a slight up-regulation instead of down-regulation was observed at 2 days of age (Fig. 2). For 65% of the genes, including Cryαa Cryγf, Cryβa1, Cryβb2, Mmp12, Tgfbi, Mfng, Col1a1, Bcl2a1, Plat, Vcam, α-Sma, and Cdh2, fold differences were higher according to qRT-PCR, indicating that microarray can underestimate the extent of gene regulation compared to qRT-PCR, while for the rest of the genes fold differences were of comparable magnitude. The expression of onco-Dbl in the transgenic mouse lenses was confirmed by qRT-PCR for all time points (data not shown). These data validate the microarray results and substantiate the ability of Dbl oncogene to modulate genes involved in lens morphogenesis, apoptosis, angiogenesis and EMT processes.

**Figure 3:**
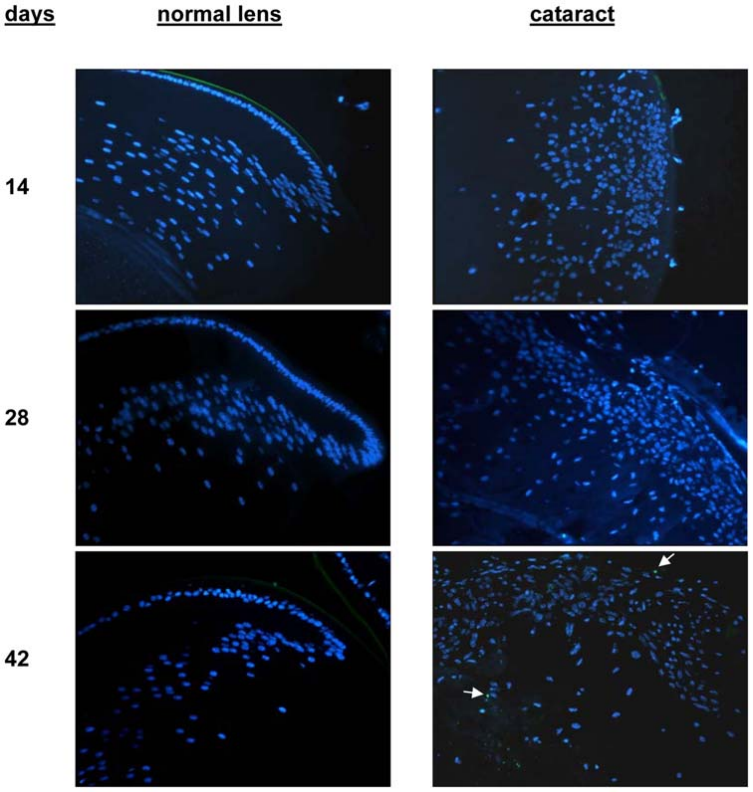
Occurrence of apoptosis in Dbl transgenic lenses. Lens sections were stained with TUNEL (green) to reveal apoptotic cells. Nuclei were stained with DAPI (blue). Sections were from normal and transgenic lenses. Eyes were collected at the indicated times. The two apoptotic cells detected at 42 days are indicated by the arrowheads.

**Figure 4:**
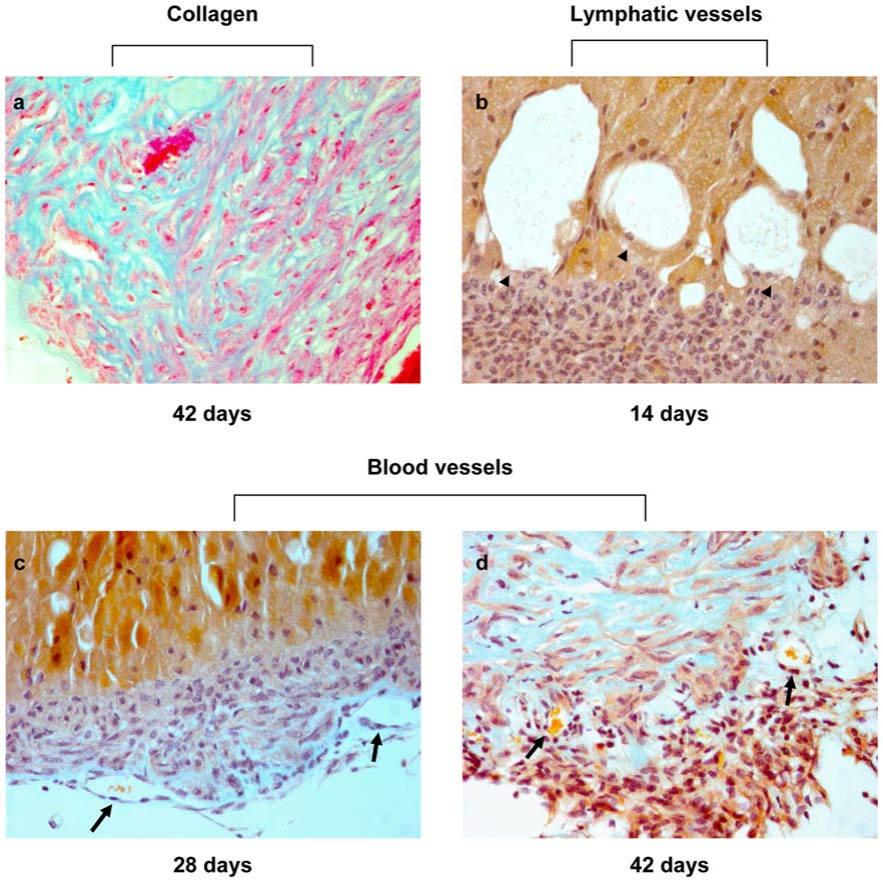
Collagen and vessel detection in Dbl transgenic lenses. Eyes were fixed with 2% paraformaldehyde and paraffin embedded. Serial sections were stained with Azan-Mallory for collagen (blue stain) or with Ignesti to reveal vessels. Arrowheads point to some of the lymphoid vessels shown and arrows indicate blood vessels. Erythrocytes are stained in orange. (a–d) 200×.

To further validate the microarray results we analyzed normal and Dbl lens sections by specific staining and immunohistochemistry. The anti-apoptotic effect of Dbl expression was assessed by end-labeling of DNA strand breaks on paraffin embedded sections of normal and transgenic lenses (TUNEL assay). Fig. 3 shows the results obtained for lenses of 14, 28 and 42 days old mice. No major differences can be seen between normal lenses and cataracts and except for a couple of cells in lenses of 42 day old mice no apoptotic event could be detected. We then evaluated the expression of collagen type 1 by staining normal and Dbl-lens sections with Azan-Mallory that stains collagen fibers in blue. By 6 weeks of age Dbl-lenses display a strong blue staining clearly demonstrating the presence of collagen type 1 (Fig. 4, panel a).

**Figure 5:**
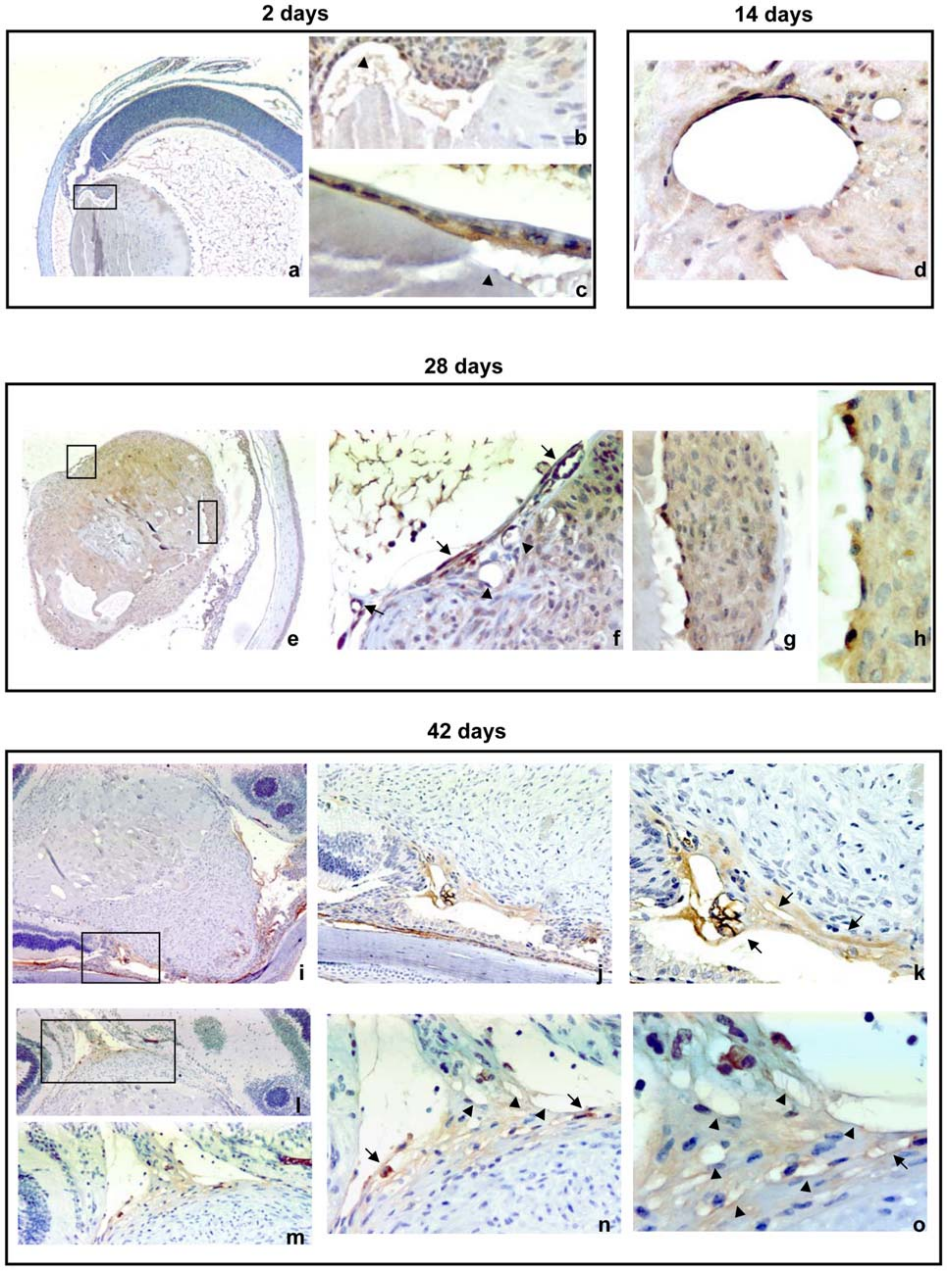
CD31 staining of vessels in Dbl transgenic lenses. Eyes collected at different times were fixed with 2% paraformaldehyde and paraffin embedded. Serial sections were treated with anti- CD31 antibody. Arrows point to blood vessels, arrowheads to lymphatic vessels. The box in a denotes the region shown at higher magnification in b and c. The boxes in e denote the regions shown at higher magnification in f, g, and h, respectively. The box in i denotes the region shown at higher magnification in k and j. The box in l denotes the region shown at higher magnification in m, n and o. (a, e, i) 40×; (k, l, m) 100×; (b, d f, g, j, n) 200× (c, h, o,)400×

Microarray results indicated that genes involved in positive regulation of angiogenesis are modulated in Dbl lenses. This was quite surprising since the lens is not vascularised, not even in pathological conditions and the embryonic lens vasculature in mice begins to degenerate after birth and is completely regressed at weaning (3 weeks). Therefore, we examined sections of normal and Dbl lenses at different times after birth for evidence of vasculature. As shown in Fig. 4, panels b–d, both lymphatic and blood vessels could be observed in Dbl lenses throughout the 6 weeks of examination. Lymphatic vessels (arrowheads) are shown in a lens section from a 14 day old mouse and blood vessels (arrows) are shown in lens sections from a 28 and a 42 day old mouse, with erythrocytes stained in orange. The vascular structure filled with red blood cells suggests the presence of a functional vasculature and indicates that the up-regulation of pro-angiogenic genes correlates with the development of vessels. The presence of vessels was confirmed by immunohistochemistry with an anti CD31 antibody. As shown in Fig. 5, lymphatic vessels (arrowheads) appear at 2 days of age and increase in number as the mice grow older (see also Fig. 4, panels b–d). Blood vessels (arrows) can be seen at 28 days of age, increase in number by 42 days of age and are mostly localized at the periphery of the lens.

**Figure 6:**
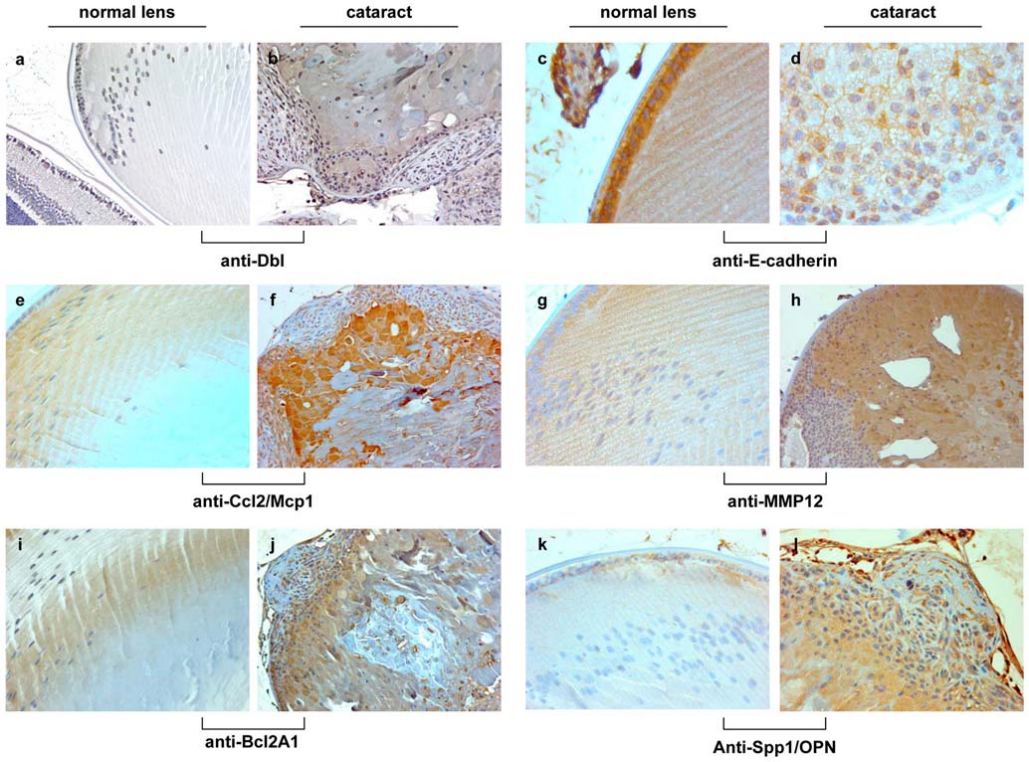
Immunohistochemistry analysis of Dbl transgenic lenses. Eyes were fixed with 2% paraformaldehyde and paraffin embedded. Serial sections were treated in a microwave oven four times with citrate buffer (pH 6.0) for 5 min at 960 W. Sections were saturated with 10% BSA in PBS with 0.1% Triton X-100 and incubated overnight at 4°C in a humidified chamber with the specific primary antibodies. Primary antibodies included: anti-Dbl rabbit polyclonal antibody (Santa Cruz Biotechnology) (a and b), anti E-cadherin mouse monoclonal [21], c and d; anti Ccl2/MCP-1 rabbit polyclonal (Abcam), e and f; anti MMP12 rabbit monoclonal (Novus Biologicals), g and h; anti Bcl2A1 rabbit polyclonal (Abcam), i and j; anti SPP-1 mouse monoclonal (Santa Cruz Biotechnology), k and l. The reactions were developed with LSAB2 System-HRP (Dako). (g, h) lenses from 14 day old mice; (c, d, k, l) lenses from 28 day old mice; (a, b, e, f, i, j) lenses from 42 day old mice. (a, b, f, h, j,) 100×; (e, g, i, k, l) 200×; (c, d) 400× Images were obtained with a Leica DMRB microscope. Micrographs were taken on LEICA DFC 320 camera.

Finally, we confirmed the expression of onco-Dbl in transgenic lenses (Fig. 6, a–b) and analyzed by immunohistochemistry the expression of E-Cadherin, a marker of epithelial cells, MMP12, whose expression causes the breakdown of basement membrane components, Ccl2/MCP-1, a potent inducer of angiogenesis, and Bcl2a1 and Spp1, two anti-apoptotic proteins (Fig. 6). Paralleling the expression patterns observed with the cDNA microarray and RT-PCR analysis, the sections of Dbl-lenses demonstrated significant reduction in E-cadherin expression in comparison with normal lens epithelial monolayer, heavily and specifically stained by the anti-E-cadherin antibody (Fig. 6, c–d). Conversely, immunoreactivity for Ccl2/MCP-1 (Fig. 6, e–f), MMP12 (Fig. 6, g–h), Bcl2A1 (Fig. 6, i–j), and SPP-1 (Fig. 6, k–l) can be observed in the Dbl-lenses while normal lenses were negative or showed only faint and probably non-specific immunoreactivity. Taken together our results indicate that Dbl oncogene expression in the lens can cause modulation of genes involved in the EMT process and angiogenesis.

## Discussion

Deficiencies in specific Dbl proteins usually do not induce major developmental abnormalities as revealed by studies with mice knock-out for specific Rho GEFs, even though in a few cases lack of GEFs can cause embryonic lethality and/or association with abnormal development. A likely explanation for the absent or subtle phenotypes in GEF-null mice is redundancy or compensation. Moreover, only a few GEFs have been found mutated in human cancer despite the fact that many of them have been originally isolated as activated, highly transforming oncogenes. Similarly, Dbl-null mice show subtle developmental abnormalities [27] and attempts to induce tumors in onco-Dbl transgenic mice could be achieved only in the absence of a functional p53 [1].

The analysis of the animal model described here indicates that onco-Dbl expression can induce major abnormalities even if such defects are restricted to the lens tissue. Using microarray to determine changes in gene expression associated with the abnormalities detected we observed strong down-regulation of expression of most of the genes responsible for eye development and lens structure and function [28]. For several of them the down-regulation increased with time. Crystallins are the main structural proteins of the lens, while filensin, a member of the intermediate filaments protein superfamily exclusively expressed in the eye lens, is essential for lens function, and specifically contributes to the optical properties of the lens by maintaining the three dimensional architecture of lens fiber cells. Thus, onco-Dbl deeply affects lens development and structure by strongly altering the expression of lens specific genes.

Epithelial cells are characterized by a polarized morphology. Wnt, Notch, and TGFβ/bone morphogenetic protein (Bmp) signaling networks are implicated in the maintenance of tissue homeostasis and are transduced to the canonical pathway for cell fate determination, and to the non canonical pathway for control of cell movement and tissue polarity [24]. We have found that genes involved in the Wnt, Notch and TGFβ signaling are modulated in Dbl-lenses. No genes encoding for TGFβ or TGFβ receptors were up-regulated and Smad genes are unaffected or down regulated (Smad1 and Smad5). On the other hand, Bmp1 and its receptor, Bmpr1a, as well as the fibroblast growth factor receptor 1 (Fgfr1) are up regulated in Dbl-lenses. The significance for such modulation is not clear but it is possible that the Wnt, Notch and TGFβ/Bmp signaling are perturbed in the Dbl-lenses for stimulation of the non-canonical pathway for the activation of RhoA and JNK signaling cascade to control cell movement and tissue polarity.

Pro-apoptotic genes were down-regulated in Dbl-lenses. On the other hand, genes encoding proteins with anti-apoptotic activity, like Bcl2a1 and osteopontin were highly up-regulated. Bcl2, the first apoptotic regulator identified through its involvement in the t14;18 chromosome translocation that hallmarks follicular lymphoma, acts by promoting cell survival [29], [30] and osteopontin stimulates expression of anti apoptotic proteins [31], [32]. Our data confirmed that no apoptosis is occurring in the Dbl transgenic mouse lenses (see Fig. 3) and support a role for Dbl in promoting cell survival through the modulation of genes regulating apoptosis.

Our observations that Dbl modulates genes involved in cell adhesion, cell polarity and apoptosis suggest that onco-Dbl expression in mouse lenses induces events associated with EMT, the process by which epithelial cells loose their characteristics, such as cell–cell adhesion, polarity, and lack of motility, and acquire mesenchymal features, including motility, invasiveness and resistance to apoptosis. In EMT the expression of epithelial proteins is suppressed and expression of mesenchymal genes is enhanced [12]–[14]. Likewise, we found that expression of E-cadherin (Cdh1) is down regulated in Dbl-lenses while N-cadherin (Cdh2) is up-regulated. In addition, the expression of the fibroblast marker α-Sma (Αsma) and of Ki-67, a marker of cycling cells as well as EMT, was significantly up-regulated in Dbl-lenses. Among the genes identified as possible players of EMT collagen IV, the major structural component of basal membrane, was down regulated, while collagen I was up-regulated. Moreover, Dbl expression induces changes in the tight-junction protein ZO-1, thus probably affecting epithelial cell polarity, and strong up-regulation of MMP12, which is involved in the proteolysis of extracellular matrix [33]. Periostin (Postn), an osteoblast-specific factor, and Tgfbi, a structural homolog of periostin, were also up-regulated in Dbl-lenses. TGFBI is a secreted ECM protein mainly expressed in fibroblasts, keratinocytes, and muscle cells and is involved in cell–matrix interaction and cell migration. Finally, as discussed above, several genes involved in the inhibition of apoptosis were also up regulated. Collectively, these data indicate that the expression of Dbl alone is sufficient to confer EMT to lens epithelial cells. It should be noted, however, that not all the genes considered hallmarks of the EMT process, such as vimentin, were found modulated in the Dbl-lenses. Many different, often crosstalking mechanisms cause EMT which comprises a wide spectrum of changes in epithelial plasticity. These may involve less or more severe gene expression changes toward a mesenchymal cell phenotype. Moreover, the fibroblastoid phenotype expressed may be different from one epithelial cell type to another and the expression of epithelial and mesenchymal molecules may not always be comparable in different tissues in vivo.

Examination of transgenic lenses indicated that onco-Dbl expression can promote neovascularisation. In fact, both lymphangiogenesis and angiogenesis can be observed in the lenses of Dbl transgenic mice throughout the 6 weeks of examination. These observations imply that onco-Dbl induces expression of pro-angiogenic factors and inhibits antiangiogenic protein expression. Microarray analysis confirmed the modulation of genes involved in angiogenesis. Annexin II, for example, stimulates cell proliferation and angiogenesis and is a putative receptor for tissue-type plasminogen activator (Tpa/Plat), another gene activated in Dbl-lenses, which generates plasmin, known to promote angiogenesis [34]. Endoglin and neuropilin 1 were also up-regulated in Dbl-lenses. Endoglin (CD105) is predominantly expressed on cellular lineages within the vascular system and is highly expressed on endothelial cells during tumor angiogenesis and inflammation [35]. Neuropilins (Nrp1 and Nrp2) are multifunctional receptors which are expressed on normal vascular smooth muscle and endothelial cells. Finally, another pro-angiogenic factor which is highly up-regulated in Dbl-lenses is the monocyte chemoattractant protein-1 (Ccl2/Mcp-1). Indeed, in addition to VEGF or other well validated angiogenic factors, chemokines have also been shown to be potent angiogenic factors and Ccl2 has been reported to be a potent inducer of angiogenesis [23].

Some of the genes modulated in Dbl-lenses have been implicated with the transformation of lens epithelial cells to mesenchymal cells in anterior subcapsular cataracts and posterior capsule opacification, both of which are secondary cataracts formed from residual lens epithelial cells after cataract surgery [36]. These cataracts appear to be accompanied by epithelial cell proliferation, synthesis of the fibroblast marker α-Sma, and accumulation of abnormal extracellular proteins, including type I and type III collagen. TGFβ and its signalling pathway are activated and considered the causative factor of lens cell transdifferentiation observed in these cataracts [37].

The results we have obtained are not compatible with Dbl inducing anterior subcapsular cataracts or posterior capsule opacification for several reasons. As already discussed above, no genes encoding for TGFβ or TGFβ receptors were found up-regulated in Dbl-lenses and the Smad genes were not modulated or were significantly down regulated. On the other hand, genes which are highly up-regulated in Dbl-lenses, such as Mmp12, Ccl2, and Spp1, have not been described in EMT occurring in these cataracts. In addition, we identified up-regulation of genes that are involved in resistance to apoptosis and induction of angiogenesis, while apoptotic cell death and decreased expression of Bcl-2 occur in lens epithelial cells in anterior polar cataracts [38] and angiogenesis has never been described as a pathologic consequence of cataract or lens injury. Thus, Dbl oncogene seems to induce alterations in the lenses that are significantly different from those occurring in anterior subcapsular cataracts and posterior capsule opacification.

The reason why Dbl oncogene expression affects only lens development and lens epithelial cell differentiation is not clear, since the oncogene is regulated by the metallothionein promoter sequences, a housekeeping promoter that, in transgenic mice, is known to promote expression of exogenous proteins in a variety of cell types. In addition, Dbl oncogene transforming activity has never been reported for epithelial cells. One possible explanation can be that the lens resides in a hypoxic environment and metallothionein gene expression is induced by a variety of stimuli, such as metal exposure, oxidative stress, glucocorticoids, and also by hypoxia [39]–[42]. Thus, the hypoxic condition of the lens may favor the expression of onco-Dbl in this tissue. Moreover, the lens is a rather peculiar, unique structure of the organism, being constituted only by epithelial cells present at different stages of maturation and differentiation. Thus, the lens may constitute the right cellular environment for a strong and effective Dbl oncogene expression.

In conclusion, while some of the alterations induced by Dbl oncogene expression in mouse lenses resemble in part those described for certain types of cataracts, the extent of the abnormalities, the modulation of certain genes and the block of the apoptotic pathway together with the induction of lymphangiogenesis and vascularization in the lens suggest that the expression of onco Dbl in epithelial cells may cause changes that cannot be merely ascribed to a fibrotic process. While we cannot describe the Dbl-induced alterations in the lens as a neoplastic event we may speculate that they represent morphological changes indicative of a predisposition for malignancy. Moreover, to our knowledge this is the first observation that a GEF may be directly responsible for induction of vascularization of an epithelial tissue.

## Supporting Information

Table S1 Primer pairs used for real-time quantitative RT-PCR. All primer pairs were designed using Primer-3 software from sequences in GenBank with a Tm optimum of 60°C and a product length of 80-150 nt. The * indicates the reference genes used for data normalization.
(0.59 M DOC)
