# Supplementary material for: Induction of Epithelial Mesenchimal Transition and Vasculogenesis in the Lenses of Dbl Oncogene Transgenic Mice
Source: PLoS One. 2009 Sep 16;4(9):e7058. doi: 10.1371/journal.pone.0007058 (PMC2739440; doi:10.1371/journal.pone.0007058)
Supplement: Table S1 — Primer pairs used for real-time quantitative RT-PCR. All primer pairs were designed using Primer-3 software from sequences in GenBank with a Tm optimum of 60°C and a product length of 80-150 nt. The * indicates the reference genes used for data normalization. (0.59 M DOC) [file pone.0007058.s001.doc]

Table S1. Primer pairs used for real-time quantitative RT-PCR.

| **Gene** | **Primers** |
| --- | --- |
| smooth muscle alpha-actin (Asma) | Forward 5'-tgtgctggactctggagatg-3' |
|  | Reverse 5'-atgtcacggacaatctcacg-3' |
| B-cell leukemia/lymphoma 2 related protein A1 (Bcl2a1) | Forward 5'-ctggatccatggctgagtctgagctcatgta-3' |
|  | Reverse 5'-cggtcgacttacttgaggagaaagagca-3' |
| beaded filament structural protein in lens-CP94 (Bfsp1) | Forward 5'-agacgctgaggaatgagctaga-3' |
|  | Reverse 5'-ctgtctcggtttagctgctgta-3' |
| bone morphogenetic protein 1 (Bmp1 ) | Forward 5'-gaaggagtgtacgtgggctatc-3' |
|  | Reverse 5'-ggcttcttactgccacagaatc-3' |
| chemokine (C-C motif) ligand 2 (Ccl2) | Forward 5'-cactcacctgctgctactcatt-3' |
|  | Reverse 5'-gtatgtctggacccattccttc-3' |
| E-cadherin (Cdh1) | Forward 5'-ctaccaaagtgacgctgaagtc-3' |
|  | Reverse 5'-acccagtctcgtttctgtcttc-3' |
| N-cadherin (Cdh2) | Forward 5'-cgcgtttgatcttcccttatc-3' |
|  | Reverse 5'-gcaggatggaaatgttggac-3' |
| collagen, type I, alpha 1 (Col1a1) | Forward 5'-aaagacggactcaacggtctc-3' |
|  | Reverse 5'-gcaggaagctgaagtcataacc-3' |
| collagen, type IV, alpha 1 (Col4a1) | Forward 5'-ctcactgtggatcggctattc-3' |
|  | Reverse 5'-gcgcttctaaactcttccagac-3' |
| collagen, type IV, alpha 2 (Col4a2) | Forward 5'-gtgtctgctgttccagggttc-3' |
|  | Reverse 5'-aggtagccgatgctcacact-3' |
| crystallin, alpha B (Cryab) | Forward 5'-cctgttggagtctgacctcttc-3' |
|  | Reverse 5'-cccagaaccttgactttgagtt-3' |
| crystallin, beta A1 (Cryba1) | Forward 5'-gggagcaatgcctatcatattg-3' |
|  | Reverse 5'-tggcgtccaataaagttctctt-3' |
| crystallin, beta B2 (Crybb2) | Forward 5'-gacagactccctcagctctctg-3' |
|  | Reverse 5'-gcacatcgtcgtctacaatctc-3' |
| dynactin 2 (Dctn2*) | Forward 5'-gttctctcaagctgccaaagtt-3' |
|  | Reverse 5'-gcaacagttctaccgtttccat-3' |
| formin homology 2 domain containing 3 (Fhos2) | Forward 5'-cagtgagtttgccctggagtat-3' |
|  | Reverse 5'-tcagcatagctcagaccctgt-3' |
| glutathione peroxidase 1 (Gpx1*) | Forward 5'-ctacaccgagatgaacgatctg-3' |
|  | Reverse 5'-cattcacttcgcacttctcaaa-3' |
| antigen identified by monoclonal antibody Ki 67 (Ki-67) | Forward 5'-aaagatgcccgacaaatctc-3' |
|  | Reverse 5'-ggttcctcctgccagttaaac-3' |
| O-fucosylpeptide 3-beta-N-acetylglucosaminyltransferase (Mfng) | Forward 5'-atccacgcctctgagctg-3' |
|  | Reverse 5'-agttggcggttgatgcag-3' |
| major intrinsic protein of eye lens fiber (Mip) | Forward 5'-gctgtccgaggaaacctagc-3' |
|  | Reverse 5'-gcctctcgtcgtatgtagcaa-3' |
| matrix metallopeptidase 12 (Mmp12) | Forward 5'-gagtccagccaccaacattact-3' |
|  | Reverse 5'-atatgctcctgggatagtgtgg-3' |
| plasminogen activator, tissue (Plat) | Forward 5'-ctggaatagcagtgttctgtcg-3' |
|  | Reverse 5'-ccctgccttaaagacatagcac-3' |
| presenilin 1 (Psen1*) | Forward 5'-gcagcaggcgtatctcattat-3' |
|  | Reverse 5'-tgtttcaaccagcatacgaagt-3' |
| secreted phosphoprotein 1 (Spp1) | Forward 5'-ggtgatagcttggcttatggac-3' |
|  | Reverse 5'-ccttagactcaccgctcttcat-3' |
| transforming growth factor, beta induced (Tgfbi) | Forward 5'-caacggtgtcattcatttcatt-3' |
|  | Reverse 5'-cagcttgtttgaggatgtcaat-3' |
| vascular cell adhesion molecule 1 (Vcam1) | Forward 5'-ccgagctaaattacacattgacc-3' |
|  | Reverse 5'-ccttgtggagggatgtacagag-3' |
